# Characteristics and Functions of Different Intestinal Segments in Juvenile Greater Amberjack (*Seriola dumerili*)

**DOI:** 10.3390/ani15111672

**Published:** 2025-06-05

**Authors:** Kunfeng Zhu, Mouyan Jiang, Mengyao Yan, Yang Huang, Tonglin Yang, Chunhua Zhu

**Affiliations:** 1 Fisheries College, Guangdong Ocean University, Zhanjiang 524088, China; 2112101067@stu.gdou.edu.cn (K.Z.); jiangmouyan@gdou.edu.cn (M.J.); mengyao8085@163.com (M.Y.); zjouhy@126.com (Y.H.); ytl990617@163.com (T.Y.); 2 Guangdong Research Center on Reproductive Control and Breeding Technology of Indigenous Valuable Fish Species, Guangdong Ocean University, Zhanjiang 524088, China; 3 Guangdong Provincial Key Laboratory of Aquatic Animal Disease Control and Healthy Culture, Guangdong Ocean University, Zhanjiang 524088, China; 4 Development and Research Center for Biological Marine Resources, Southern Marine Science and Engineering Guangdong Laboratory (Zhanjiang), Zhanjiang 524088, China; 5 Agro-Tech Extension Center of Guangdong Province, Guangzhou 510520, China

**Keywords:** greater amberjack (*Seriola dumerili*), intestine, digestion and absorption, immune, microbiome

## Abstract

The greater amberjack (*Seriola dumerili*), a key species in marine aquaculture, relies heavily on its intestine for nutrient absorption and immune function. However, the structural and functional specialization of its intestinal segments remains poorly understood. In this study, we divided the intestine of *S. dumerili* into foregut, midgut, and hindgut, and conducted a multi-omics analysis integrating histological staining (H&E/AB-PAS), digestive enzyme assays, transcriptome sequencing, and 16S rRNA microbiota profiling to characterize structural, functional, molecular, and microbial differences across intestinal segments. Histological examinations revealed that brush border microvillus length, muscle layer thickness, and folding height were significantly greater in the foregut and hindgut compared to the midgut, while mucus and goblet cell density was higher in the foregut and midgut. Digestive enzyme assays showed that lipase activity peaked in the foregut, α-amylase in the midgut, and protease in the midgut and hindgut. Alkaline phosphatase (AKP) and acid phosphatase (ACP) activities were highest in the foregut and midgut. Immune-related enzyme activities (SOD (Superoxide dismutase), GSH-Px (Glutathione peroxidase), T-AOC (Total Antioxidant Capacity)) were elevated and MDA levels were lower in the midgut, indicating its role as the primary immune site. Transcriptome analysis identified segment-specific expression of nutrient transporters, such as *slc6a19b* (hindgut, protein), *apoa1b* (foregut, lipid), and *slc37a4* (midgut, carbohydrate). Microbiome analysis revealed *Ruminococcus* dominance in the foregut (lipid digestion) and *Prevotella*, *Bifidobacterium*, and *Lactobacillus* enrichment in the midgut (carbohydrate metabolism and immunity). These findings highlight functional zonation in *S. dumerili*: the foregut specializes in lipid digestion, the midgut in carbohydrate metabolism and immunity, and the hindgut in protein digestion. This study provides foundational insights for optimizing aquaculture practices and advancing research in nutrition, immunology, and disease modeling in *S. dumerili*.

## Simple Summary

The greater amberjack, a valuable farmed fish, uses its intestines for both nutrient absorption and immune defense. However, how different parts of its gut specialize in these roles remains unclear. We studied the foregut, midgut, and hindgut sections of its intestine to understand their unique features. The foregut and hindgut showed stronger structures for processing food, while the midgut had more mucus-producing cells. Tests revealed the foregut works best at digesting fats, the hindgut handles carbohydrates and demonstrates the strongest immune capacity, and the hindgut helps break down proteins. Genetic analysis further validates the functions of each intestinal segment and identifies specific genes used by each segment for transporting nutrients. We also found distinct helpful bacteria in each area—fat-digesting microbes dominated the foregut, while carbohydrate-processing and immunity defense bacteria thrived in the midgut. These discoveries show the fish’s gut has specialized zones: the foregut for fats, the midgut for carbs and immunity defense, and the hindgut for proteins. Understanding this “division of labor” helps fish farmers design better diets and health strategies for amberjacks.

## 1. Introduction

The intestine serves as a key organ for fish, directly linked to essential life processes such as fish growth, development, reproduction, and immunity [1]. Therefore, comprehending the structure and function of fish intestines is crucial for maintaining gut homeostasis in aquaculture settings, optimizing compound feed formulations [2] to match segment-specific digestive capacities [3] (lipid-rich diets for foregut efficiency), and developing targeted strategies to enhance intestinal immunity (probiotics for midgut mucosal protection). These insights are particularly vital for carnivorous species like greater amberjack, where digestive specialization directly impacts feed utilization and disease resistance. Over the past few decades, numerous studies have focused on the morphological and histological characteristics of fish intestines and their effects on growth, development, and reproduction [4,5,6]. The fish intestine is typically divided into three segments: the foregut, midgut, and hindgut [1,7], a classification widely adopted in teleost research to characterize regional specializations in digestion (lipid hydrolysis in foregut [5], immune surveillance in midgut [8], and protein absorption in hindgut [9], based on anatomical landmarks (intestinal flexures) and functional specializations in digestion, absorption, and immunity. This segmentation is critical for understanding regional adaptations, such as the foregut’s role in lipid hydrolysis, the midgut’s immune surveillance, and the hindgut’s protein absorption, which are essential for optimizing aquaculture diets and health management [10,11,12]. However, research on segment-specific functions in carnivorous marine fish, particularly regarding nutrient absorption and segmental immune responses, remains limited and sometimes controversial. While studies in omnivorous species (such as common carp [13]) have identified midgut dominance in carbohydrate metabolism, comparable insights into carnivorous taxa like *Seriola dumerili* are scarce. For instance, the foregut is often considered the primary site for nutrient digestion and absorption, particularly for lipids and carbohydrates, due to the presence of digestive enzymes and well-developed villi [5,6]. In contrast, the midgut, with its extensive folds and brush border microvillus, is believed to complement the foregut’s digestive and absorptive functions, particularly for macromolecules [7]. The hindgut contributes to immune defense through multifaceted mechanisms, primarily mediated by mucosal barrier functions—including mucus secretion, antimicrobial peptide production, and microbial regulation—and secondarily by structural adaptations that enhance luminal clearance. Mucus-secreting goblet cells, abundant in the hindgut epithelium, secrete glycoconjugates that form a physical barrier against pathogens while supporting symbiotic microbial communities [5]. Concurrently, the thickened muscular layers of the hindgut, particularly in species like the Amur catfish (*Silurus asotus*), enhance peristaltic efficiency, reducing pathogen residence time and mechanically disrupting microbial aggregates, which indirectly supports intestinal immune homeostasis [14]. In bony fish larvae, the hindgut also plays a specialized role in protein absorption via phagocytosis, a process linked to its unique histological features, such as expanded epithelial surface area and specialized absorptive cells [9]. This dual functionality—combining immune defense through mucosal-microbial interactions and mechanical clearance with nutrient absorption—highlights the hindgut’s adaptive significance in fish physiology. Studies in *Colossoma macropomum* have shown that proteolytic activities in the posterior intestine, supported by muscular contractions, facilitate both protein digestion and pathogen exclusion, underscoring the interplay between structural adaptations and immune-nutritional roles [9].

Recent advances further emphasize the hindgut’s role in immune-nutritional crosstalk. For instance, the hindgut mucosal barrier integrates physical (mucus layer), biochemical (antimicrobial peptides like piscidin1), and immunological (IgT-secreting plasma cells) components to neutralize pathogens while maintaining microbial homeostasis [15,16]. Microbiota-derived short-chain fatty acids (SCFAs) in the hindgut, such as butyrate, have been shown to enhance epithelial integrity and suppress pro-inflammatory pathways in teleosts [17]. Additionally, the hindgut’s capacity to modulate systemic immunity via cytokine signaling (IL-22-mediated inflammation) underscores its role as a dynamic immunological hub [18].

Goblet cells in the hindgut are critical for immune defense, with their density and mucin secretion patterns varying along the intestinal tract. In *Seriola dumerili* and related species, goblet cells are most abundant in the posterior intestine, where they secrete acidic and neutral mucins to form a protective gel layer that traps pathogens and supports symbiotic microbiota [5,19]. Studies in catfish (*Silurus asotus*) demonstrate that goblet cells differentiate from basal epithelial layers to the mucosal surface, with posterior segments exhibiting higher concentrations of acidophilic mucin-secreting cells, which correlate with enhanced antimicrobial activity [14]. These cells also interact with gut microbiota, with their mucus providing a niche for beneficial bacteria while inhibiting pathogen adhesion [17].

In terms of intestinal immunity, research has primarily focused on components such as intestinal mucus [20], epithelial cells, gut-associated lymphoid tissue [21], lysozyme, and antibodies [22]. The hindgut’s structural adaptations, such as a notably thickened muscular layer and increased goblet cell density in Amur catfish (*Silurus asotus*), suggest its critical role in immune defense, likely through enhanced mucus secretion and mechanical clearance of pathogens [14]. While studies on diverse feed formulations often focus on the midgut or foregut—such as in common carp (*Cyprinus carpio*) [13] and southern catfish (*Silurus meridionalis*) [19]—the functional specialization of intestinal segments in carnivorous species remains understudied. For example, in turbot (*Scophthalmus maximus*), dietary shifts impact lipid metabolism and antioxidant capacity in the foregut, highlighting regional differences in nutrient processing [6]. Comparative histology reveals that goblet cells in the hindgut of *S*. *asotus* differentiate from basal epithelial layers to the mucosal surface, secreting acidophilic mucins that correlate with antimicrobial activity, reinforcing its role as an immune-responsive site [14,19].

Recent work in greater amberjack (*Seriola dumerili*) links hindgut mucosal gene expression to hypoxia tolerance, showing that differential regulation of immune-related pathways in the posterior intestine influences stress resistance [23]. However, unlike omnivorous or herbivorous species, carnivorous fish like *Seriola dumerili* exhibit unique hindgut adaptations, such as elevated short-chain fatty acid metabolism, which may modulate microbial interactions and immune homeostasis [4]. Studies in rainbow trout *(Oncorhynchus mykiss*) [24] further demonstrate that foregut and midgut segments play distinct roles in digestive enzyme activity and mucosal immunity, underscoring the need for segment-specific analyses in carnivorous taxa [22]. Despite these advances, a comprehensive understanding of how intestinal regionalization influences immune function and nutrient assimilation in carnivorous fish, particularly in response to dietary changes, remains an important research gap. Greater amberjack (*Seriola dumerili*), a carnivorous marine fish, has garnered significant attention in recent years due to its rapid growth, environmental adaptability, and high market value [25]. In its natural habitat, *S. dumerili* primarily preys on small fish, crustaceans, and cephalopods, which influences its digestive physiology and intestinal structure [26]. Current research on *S. dumerili* predominantly revolves around individual development [27], hypoxia tolerance [23], growth [28], feed substitution strategies [29], and gonadal development characteristics [30]. However, there is a notable lack of studies addressing the functional differences along its intestinal tract, particularly in relation to its carnivorous diet.

While prior studies have characterized digestive enzyme activities and mucosal immunity in teleosts [6,22], the physiological adaptations of *Seriola dumerili*—a key carnivorous species in aquaculture—remain understudied. Specifically, how its intestinal segments coordinate nutrient assimilation, microbial interactions, and immune defense during dietary shifts is unclear. By integrating histochemical analysis of goblet cell distribution, digestive enzyme profiling, and mucosal gene expression, this study aims to deepen our understanding of *S. dumerili* physiology, particularly the role of the hindgut in lipid metabolism and immune homeostasis, which are critical for optimizing its nutritional management in intensive farming systems. Additionally, transcriptome sequencing and microbiota analysis is performed to elucidate the functional and microbial diversity across the foregut, midgut, and hindgut. The findings from this study are expected to provide valuable insights into the digestive and immune functions of *S. dumerili*, laying a solid foundation for future research in fish nutrition, immunology, disease modeling, and developmental biology.

## 2. Materials and Methods

### 2.1. Experimental Fish Sample Collection

All juvenile *S. dumerili* used in the experiments were six months old and raised in fishing rafts off the coast of Qixia Village, Dongshan County, Zhangzhou City, Fujian Province, China. The fish were fed twice daily (at 9:00 and 15:00) with a diet of fresh mixed fish, including anchovies (*Engraulis japonicus*), sardines (*Sardinops sagax*), and mackerel (*Scomber japonicus*), to mimic their natural diet. Water quality parameters were monitored daily to maintain dissolved oxygen levels between 5.9 and 8.7 mg/L, nitrite levels below 0.01 mg/L, salinity fluctuations within 0.15, a constant pH of 8.5, and ammonia nitrogen levels below 0.02 mg/L. Before sampling, the fish underwent a 24 h fasting period to ensure complete evacuation of food residues from the intestines. Eighteen fish were randomly selected for the experiment and sedated with 2-methoxy-4-(2-propenyl) phenol (Sigma, St. Louis, MI, USA). The average body length was 24.7 ± 1.46 cm, total length was 29.73 ± 1.58 cm, and weight was 388.8 ± 45.07 g. The length of the intestine was measured at 20.1 ± 2.67 cm, and the specific intestinal length ratio was calculated as 0.8 ± 0.08 (mean ± SE (*n* = 18).

**Figure 1:**
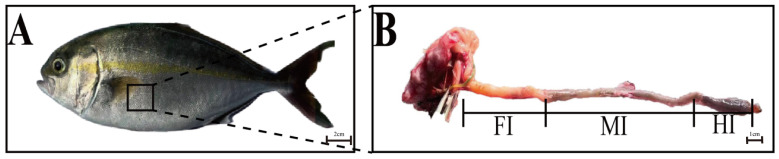
(**A**) schematic diagram of the digestive tract of a juvenile *S. dumerili*. (**A**), The experimental fish; (**B**), Schematic diagram of different segments of the intestine. FI: Anterior intestine; MI: Middle intestine; HI: Posterior intestine.

After dissection, the intestines were gently flushed with ice-cold phosphate-buffered saline (PBS, pH 7.4) [31] using a syringe until the effluent was clear to remove any residual food content. The intestines were divided into foregut, midgut, and hindgut (Figure 1) using anatomical landmarks (first/second flexures) [25], following the segmentation protocol established in juvenile greater amberjack by Navarro-Guillén et al. [25] and validated in related species like common carp [13]: Foregut refers to the anterior segment, extending from the pyloric sphincter to the first intestinal flexure, characterized by a thick muscularis propria for peristalsis; Midgut refers to the middle segment between the first and second flexures, rich in goblet cells and immune-related enzymes; Hindgut refers to the posterior segment from the second flexure to the anus, featuring tall intestinal folds for chyme retention [25]. Each section was subdivided into four parts: one preserved in Bouin’s solution for hematoxylin and eosin (H&E) staining, another preserved in Carnoy’s Fluid for Alcian blue-periodic acid-Schiff’s reagent (AB-PAS) staining, a third part stored in liquid nitrogen for microbial analysis and transcriptome sequencing, and the final part stored at −80 °C for enzyme activity testing and RT-qPCR analysis. All procedures were conducted in accordance with the guidelines set forth by the Animal Protection and Use Committee at Guangdong Ocean University (protocol number: GDOU-IACUC-2022-A0925).

### 2.2. H&E Staining and AB-PAS Staining of the Intestine

The intestinal specimens were immersed in Bouin’s solution and fixed for 24 h. They were then dehydrated using alcohol gradients, cleared with xylene, and embedded in paraffin blocks. Three intestinal samples of each segment (n = 18 fish per segment) were stained with H & E and AB-PAS, and three sections of each fish at each stage were observed for intestinal morphology and mucous cell distribution. The paraffin blocks were cut into 7 μm sections using a YD-202 slicer (Jinhua Yidi Medical Equipment Co., Ltd., Jinhua, China), stained with H&E, sealed with neutral gum, and examined under a Nikon ECLIPSE Ni-E microscope (Nikon, Tokyo, Japan) equipped with a DS-Ri2 Nikon microscope (Nikon, Tokyo, Japan). Images were acquired using NIS-Elements BR 5.20.02 64-bit software (Nikon, Tokyo, Japan). Measurements of mucosal folds [32], brush border microvillus height (vertical distance from the base to the tip of the brush border microvillus), and muscle layer thickness (lateral aspect of submucosa to medial aspect of lamina propria) were performed using NIS Elements software (NiS-Elements BR 5.20.02 64-bit). Similarly, intestinal samples fixed with Carnoy’s solution were sectioned, stained with AB-PAS, and imaged using a Nikon ECLIPSE Ni-E microscope (Nikon, Tokyo, Japan). Using an optical microscope, with a magnification of 200 times, we randomly selected six non-overlapping fields of view for each sample to ensure representativeness. For each field of view, we used microscope matching software (NiS-Elements BR 5.20.02 64-bit) with a size of 500 μm × 500 μm as the counting area and carefully counted the cells manually.

### 2.3. Determination of Antioxidant-Related Indices and Digestive Enzyme Activities

Enzyme activities were measured in six biological replicates (n = 6 per segment) using homogenized samples, with values expressed as mean ± SE. Frozen samples of the foregut, midgut, and hindgut were precisely weighed and transferred to prechilled tubes. A nine-fold volume of ice-cold 0.9% NaCl buffer containing 0.1% Triton X-100 was added, and tissues were homogenized using an FastPrep-24™ 5G (MP Biomedicals, Santa Ana, CA, USA) (15 s cycles, 6.0 m/s) in an ice bath to maintain enzyme stability during disruption [13]. The homogenate was centrifuged at 3000 r/min for 15 min at 4 °C, and the supernatant was collected for analysis. Specific assays for the following parameters were conducted using Nanjing Jiancheng Bioengineering Institute kits and instruments (Nanjing, China): Lipase (LPS, A054-2-1), α-Amylase (AMS, C016-1-1), Trypsin (A080-2-2), Superoxide dismutase (SOD, A001-3-2), Glutathione peroxidase (GSH-Px, A005-1-2), Total Antioxidant Capacity (T-AOC, A015-2-1), Malondialdehyde (MDA, A003-1-2), Acid Phosphatase (ACP, A060-2-2), Alkaline Phosphatase (AKP, A059-2-2). Enzyme activities and MDA concentration were measured using a Thermo Multiskan GO 1510 microplate reader (Thermo, Waltham, MA, USA, 51119200, SN:1510-05190C) for colorimetric assays, while protein levels were determined via the bicinchoninic acid (BCA) method on a SHIMADZU UV-1800 spectrophotometer (Shimadzu, Japan Model: UV-1800, SN: 1800-20240512), following the manufacturer’s protocols. For consistency with fish tissue characteristics, all samples were diluted to 1–5% homogenate concentration to ensure absorbance values fell within the kit standard curve range (0.2–1.0 OD), as validated in prior teleost studies [4,6,13].

### 2.4. Transcriptome Sequencing Analysis

Transcriptome analyses were performed using three biological replicates per segment (*n* = 3), with library construction and sequencing following standardized protocols. Total RNA was extracted from each sample using the Tissue RNA Extraction Kit (TaKaRa, Kusatsu City, Japan) following the manufacturer’s instructions. Genomic DNA contaminants were removed, and RNA quality was assessed using an Agilent 2100 Bioanalyzer (Agilent, Santa Clara, CA, USA). The enriched mRNA was fragmented and reverse transcribed into cDNA using the NEBNext Ultra RNA Library Prep Kit for Illumina. The integrity of the cDNA was verified by 1% RNase—free agarose gel electrophoresis. The double-stranded cDNA fragments were end-repaired, an A base was added, and they were ligated to Illumina sequencing adapters prior to PCR amplification. The resulting cDNA library was sequenced by Gene Denovo Biotechnology Co. on an Illumina Novaseq 6000 platform (Illumina, San Diego, CA, USA). The sequencing reads were filtered using fastp.

Bowtie2 was employed to map the reads to the ribosomal RNA (rRNA) database, and then the rRNA-mapped reads were removed. The remaining clean reads were used for assembly and calculation of gene abundance. The reads were mapped to the reference genome (*Seriola dumerili*. v1.0. genome. fa) using HISAT2.2.4 and then assembled with StringTie v1.3.1. The clean library sequencing data were deposited in the NCBI Sequence Read Archive under Bioproject Number: PRJNA1069331 (SUB13521488). The expression abundance and variation of each transcriptional region were quantified by calculating the fragments per kilobase of transcript per million mapped reads (FPKM) with RSEM software (version 1.3.3).

Principal component analysis (PCA) was carried out using the gmodels R package (http://www.rproject.org/, accessed on 25 May 2023). For the differential expression analysis of RNAs, DESeq2 software (version 1.48.1) [33] was used to compare among three different groups, while edgeR [34] was used for pairwise sample comparisons. Genes/transcripts with a false discovery rate (FDR) below 0.05 and an absolute fold change of ≥2 were defined as differentially expressed genes/transcripts. The differentially expressed genes (DEGs) related to biological functions were filtered through Gene Ontology (GO) analysis and Kyoto Encyclopedia of Genes and Genomes (KEGG) enrichment analysis.

### 2.5. Gut Microbiota Testing

Transcriptome and microbiota analyses were performed using three biological replicates per segment (n = 3), with library construction and sequencing following standardized protocols. Microbial DNA was extracted from intestinal segments using HiPure Stool DNA Kits (Magen, Guangzhou, China). The target region of 16S rDNA/ITS/18S rDNA was amplified using specific primers with barcodes [35]. After DNA extraction, polymerase chain reaction (PCR) was used to amplify the targeted gene fragments within the 16S V1–V9 region of the DNA samples. The PCR conditions were as follows: initial denaturation at 95 °C for 5 min, followed by 30 cycles of denaturation at 95 °C for 1 min, annealing at 60 °C for 1 min, extension at 72 °C for 1 min, a final extension at 72 °C for 7 min, and ending with a holding step at 4 °C. The PCR products were extracted from 2% agarose gels, and the amplified products were then purified using AMPure XP Beads. Subsequently, the products were quantified using the ABI StepOnePlus Real-Time PCR System (Thermo Fisher Scientific, Waltham, MA, USA).

High-throughput sequencing analysis was performed on the Illumina MiSeq platform (Illumina, San Diego, CA, USA). The initial sequencing data were filtered and analyzed using FASTP 0.18.0 software [36]. Accurate biological sequences were extracted through DADA2 or Deblur denoising [37]. After removing chimeric sequences with UCHIME [38], specific tags were identified. The clean tags were then clustered into operational taxonomic units (OTUs) at ≥ 97% similarity using UPARSE software [39] (version 9.2.64). Any remaining chimeric tags were removed using the UCHIME algorithm [38] to obtain effective tags for subsequent analysis. Finally, the classification of microorganisms was determined by comparing the representative sequences with the Ribosomal Database Project RDP Classifier database through homology comparison.

### 2.6. Validation of DEGs with Quantitative Real-Time PCR (qRT-PCR)

RNA was isolated using the Tissue RNA Extraction Kit from TaKaRa according to the specified protocol. To guarantee purity, any genomic DNA contaminants were eliminated. Subsequently, cDNA was synthesized via reverse transcription. The integrity of the synthesized cDNA was verified through examination using 1.0% agarose gel electrophoresis. Subsequently, primers were designed for the *S. dumerili* genes slc1a1, slc10a2, ca7, slc15a1a, aqp10a, grtp1b, alp3, and slc30a8 using the open reading frame sequences from the NCBI database. The Primer—BLAST tool was employed for this purpose, and the primers were synthesized by Shanghai Sangong Biotechnology Co. In the experiment, the housekeeping gene, β—actin, as described by Zupa [40], was utilized. The detailed primer sequences are presented in Table S1.

For qRT-PCR, a reaction mixture of 20.0 μL was prepared, consisting of 2x SYBR Green Prc Taq HS Premix (10 μL), cDNA (1 μL), Primer F (1 μL), Primer R (1 μL), and RNase-free water (7 μL). The amplification program consisted of 40 cycles at 95°C for 20 s, 60 °C for 20 s, and 72 °C for 20 s. The relative expression levels of the target genes were determined using the 2^−ΔΔCt^ method, with normalization based on a previous transcriptome analysis.

### 2.7. Statistical Analysis

Statistical analyses, encompassing significance analysis, multiple comparisons, and correlation analysis, were executed using SPSS 26.0. The data are presented as the mean ± standard error (n = 6). A variety of diversity indices, including the Chao1 index, ACE index, Shannon index, Simpson index, Good’s coverage, and Pielou’s evenness index, were computed in QIIME [41] version 1.9.1. The PD-whole tree index was calculated with the aid of picante [42] version 1.8.2.

To evaluate group differences, a two-tailed unpaired Student’s *t*-test was employed. For multiple comparisons, one-way or two-way ANOVA followed by Tukey’s test was utilized. The gut flora sequencing data were analyzed using the R statistical software and the two-tailed Wilcoxon rank—sum test. For data analysis and graph generation, software tools such as Graph Pad Prism 9.5, Origin 2020, Insight Pro 1.4.0, SciPy (Python) 1.0.0, and the ropls R program package 1.6.2 were applied.

## 3. Results

### 3.1. Structural Characteristics of S. dumerili Intestine

**Figure 2:**
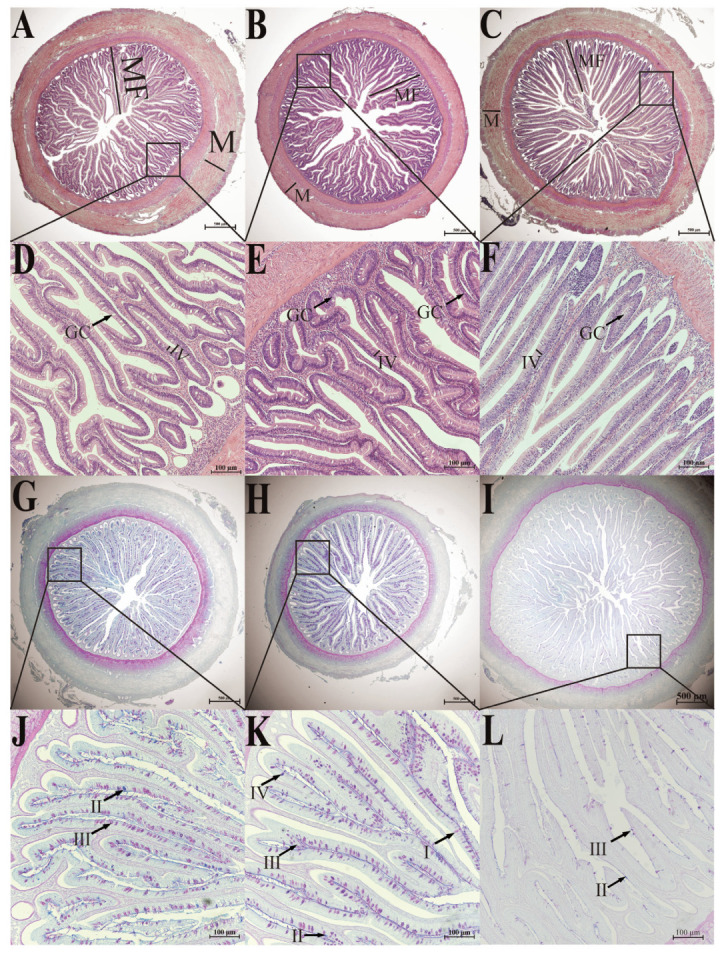
H&E staining (**A**–**F**) and AB-PAS staining (**G**–**L**) of the intestine of six-month-old *S. dumerili*. (**A**,**D**,**G**,**J)**: foregut; (**B**,**E**,**H**,**K**): midgut; (**C**,**F**,**I**,**L**): hindgut. MF: intestinal folds; M: muscularis propria; IV; brush border microvillus. GC: goblet cells, I: type I mucous cells; II: type II mucous cells; III: type III mucous cells; IV: type IV mucous cell.

**Figure 3:**
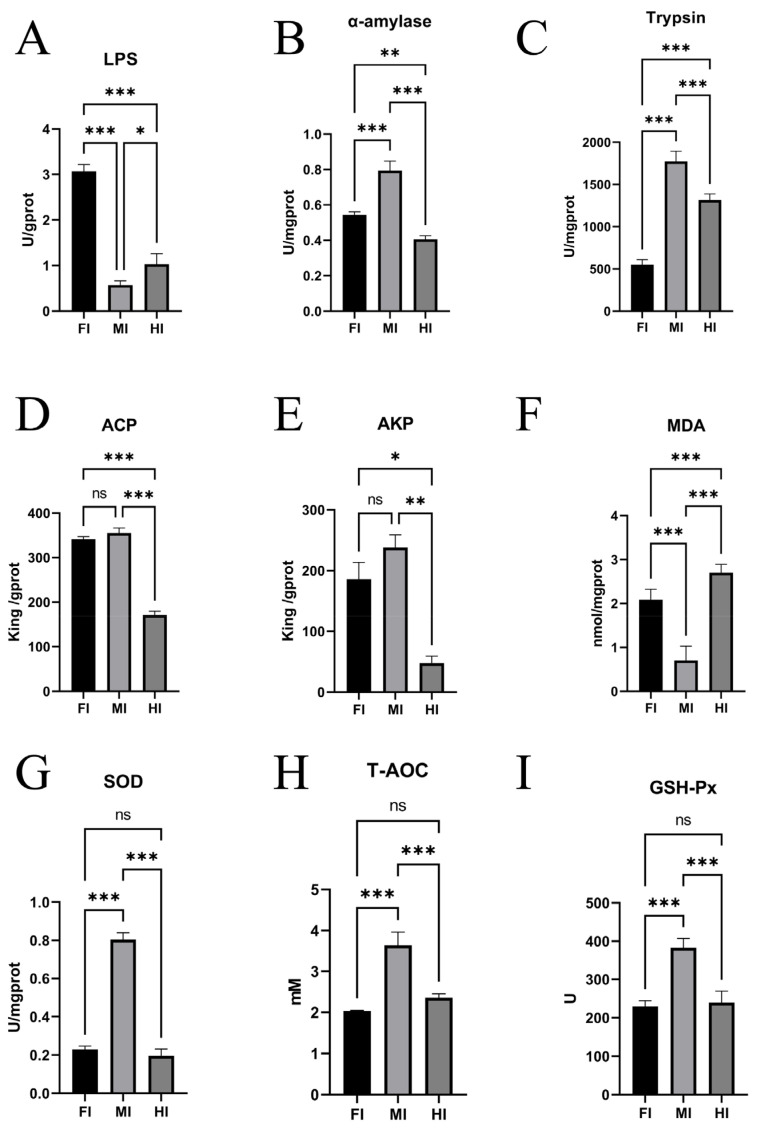
Enzyme activities and biochemical indices in different intestinal segments in *S. dumerili*. (**A**): LPS, lipase; (**B**): AMS, α-amylase; (**C**): Trypsin; (**D**): ACP, acid phosphatase; (**E**): AKP, alkaline phosphatase; (**F**): MDA, Malondialdehyde; (**G**): SOD: Superoxide dismutase; (**H**): T-AOC, Total antioxidant capacity; (**I**): GSH-Px, Glutathione peroxidase; *, *p* ≤ 0.05; **, *p* ≤ 0.01; ***, *p* ≤ 0.001; ns, *p* ≥ 0.05.

The intestinal wall of *S. dumerili* is composed of four distinct layers: the mucosal layer, submucosal layer, muscularis propria, and serosa. The intestinal wall of *S. dumerili* comprises four histologically distinct layers (Figure 2A–L), clearly visualized in H&E (Figure 2A–F) and AB-PAS (Figure 2G–L) staining. The mucosal layer extends into the intestinal lumen, forming finger-like villi composed of a single layer of columnar epithelium and lamina propria. The epithelial surface exhibits well-developed striated edges, with mucous cells, such as goblet cells, dispersed throughout the inner layer. These layers exhibit segment-specific modifications: the foregut’s thick muscularis propria supports lipid digestion (Table 1, Figure 3A), the midgut’s dense goblet cells enhance mucosal immunity (Figure 2H,K), and the hindgut’s tall folds facilitate protein absorption and fecal formation (Table 1, Figure 3C). The lamina propria primarily consists of connective tissue and lacks a distinct boundary with the submucosa. The intestinal muscular layer consists of two distinct smooth muscle sublayers: an inner circular muscle and an outer longitudinal muscle, which are critical for peristaltic movements. Between the lamina propria and submucosa, a thin lamina muscularis mucosae (mucosal muscle layer) was observed, a feature consistent with typical teleost anatomy. The outermost layer, the serosa, is a connective tissue membrane lined with mesothelial cells, distinguishing it from the adventitia found in non-serous organs such as esophagus [43]. The outer layer of the digestive tract is composed of a thin serosa, a connective tissue membrane lined with mesothelial cells, which is consistent with canonical anatomical nomenclature.

The morphology and distribution of brush border microvillus were consistent across all segments of *S. dumerili*. However, the length of villi in the foregut and hindgut did not differ significantly (*p* ≥ 0.05), but both were significantly longer than those in the midgut (*p* ≤ 0.05). The thickness of the muscularis propria varied among the segments, with the foregut being the thickest, followed by the hindgut and midgut. Similarly, the height of intestinal folds differed significantly, with the hindgut exhibiting the highest folds, followed by the foregut and midgut.

Based on the classification approach of mucous cells in crucian carps [5] and catfish [19], the intestine of *S. dumerili* contains four distinct types of mucous cells. Mucous cell types in *Seriola dumerili* intestines were distinguished by histochemical staining and carbohydrate composition (Figure 2, Table 1). Type I cells were stained red with periodic acid-Schiff (PAS), indicating the presence of neutral mucopolysaccharides. Type II cells showed blue reactivity with alcian blue (AB), confirming acidic mucopolysaccharides. Type III cells displayed a purplish-red phenotype, reflecting a matrix dominated by neutral mucopolysaccharides with minor acidic components. Type IV cells appeared bluish-purple, indicating a predominantly acidic mucopolysaccharide composition with trace neutral elements.

Intestinal distribution of mucous cells varied significantly across segments (*p* < 0.05). The midgut exhibited the highest density, followed by the foregut and hindgut. Both foregut and midgut hosted all four cell types, with Type III cells comprising the majority. These cells were predominantly situated within the mucosal epithelium, with a subset in the submucosal layer, and their volumetric density increased from the base to the apex of intestinal folds. Only blue-stained type II and purplish-red type III mucous cells were observed in multiple sections of the mucosal folds in the hindgut, and the number was much lower than that of the foregut and midgut. These cells were mainly located at the bottom of the fold (Figure 2). This indicates that the hindgut has a unique mucous cell distribution pattern. The lower number of mucous cells and their location at the bottom of the fold may contribute to a relatively less viscous mucus layer in the hindgut. This could be beneficial for fecal consolidation, as a less sticky mucus allows feces to compact more easily. Additionally, the reduced mucus secretion might be related to water reabsorption, as excessive mucus could interfere with the efficient reabsorption of water from the feces. This regional specialization suggests functional differentiation, with the midgut prioritizing mucus secretion for lubrication and epithelial protection, while the hindgut maintains a simplified mucous profile aligned with its role in waste transit and water absorption.

### 3.2. Differences in Enzyme Activities and Biochemical Indices Across Intestinal Segments

Significant differences in enzyme activities were observed among the foregut, midgut, and hindgut. Lipase (LPS) activity was highest in the foregut, followed by the hindgut and midgut (*p* ≤ 0.05) (Figure 3A). Amylase (AMS) activity was highest in the midgut, followed by the foregut and hindgut (*p* ≤ 0.05) (Figure 3B). Trypsin activity also varied significantly, with the midgut showing the highest activity, followed by the hindgut and foregut (Figure 3C). Alkaline phosphatase (AKP) and acid phosphatase (ACP) activities were similar in the foregut and midgut but significantly higher than in the hindgut (*p* ≤ 0.05) (Figure 3D,E).

In terms of antioxidant capacity, SOD activity was significantly higher in the midgut compared to the foregut and hindgut (*p* ≤ 0.05), with no significant difference between the latter two (*p* ≥ 0.05) (Figure 3G). Glutathione peroxidase (GSH-Px) activity was significantly higher in the midgut compared to the hindgut (*p* ≤ 0.05) (Figure 3I). Total antioxidant capacity (T-AOC) was also higher in the midgut than in the foregut and hindgut (*p* ≤ 0.05), with no significant difference between the latter two (*p* ≥ 0.05) (Figure 3H). Malondialdehyde (MDA) content was highest in the hindgut, followed by the foregut and midgut (*p* ≤ 0.05) (Figure 3F).

### 3.3. Transcriptome Sequencing and Differentially Expressed Genes (DEGs)

**Figure 4:**
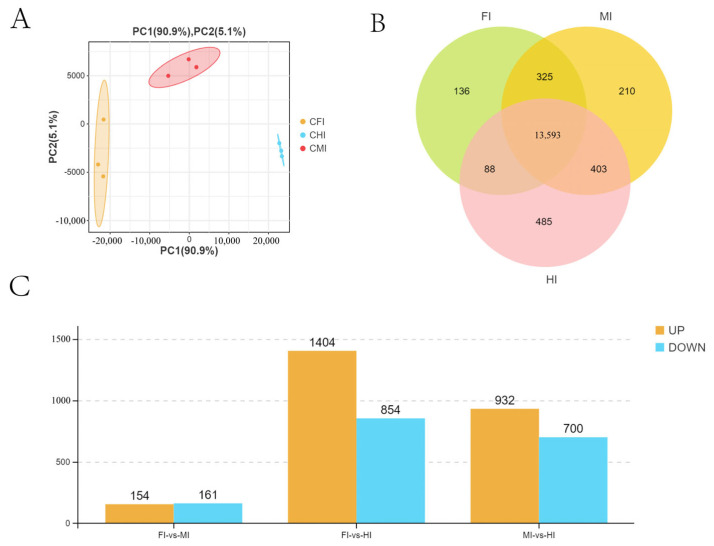
All expressed genes and DEGs obtained by RNA-seq from different intestinal segments of *S. dumerili*. (**A**) PCA showing the differences between biological groups. (**B**) Venn diagram showing the total number of genes. (**C**) DEGs obtained by RNA-seq.

A total of nine cDNA libraries were constructed and sequenced, representing the foregut (FI1, FI7, FI10), midgut (MI1, MI4, MI10), and hindgut (HI1, HI4, HI10). After quality filtering, 398,576,440 clean reads were obtained, with Q30 values exceeding 91.6% and GC content close to 50%, indicating high-quality sequencing data (Table S2). Principal Component Analysis (PCA) revealed distinct clustering of samples by intestinal segment, confirming biological replication robustness and suggesting functional differences (Figure 4A).

A total of 15,340 genes were detected, with 13,593 expressed across all segments and varying numbers unique to each segment (Figure 4B). Differential gene expression analysis identified 315 DEGs between the foregut and midgut, 2258 between the foregut and hindgut, and 1632 between the midgut and hindgut (Figure 4C).

**Figure 5:**
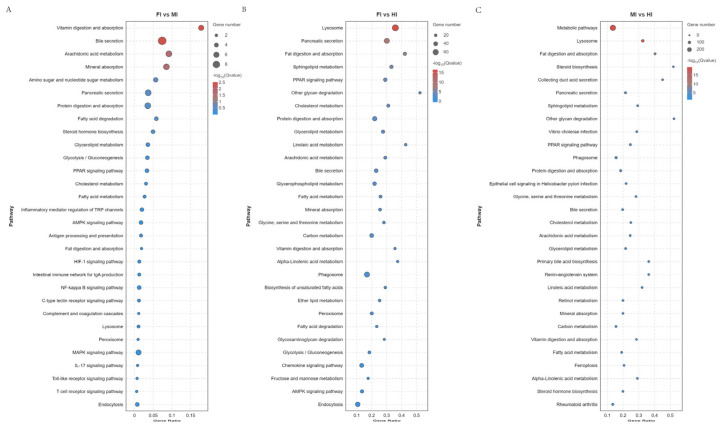
KEGG pathway enrichment analysis of the DEGs obtained by RNA-seq.(**A**) FI vs MI. (**B**) FI vs HI. (**C**) MI vs HI. The diameter of the circles represents the number of genes. The colors bar, from red to blue, represents the significance level of enrichment.

KEGG enrichment analysis revealed significant pathway differences among the segments. Specifically, 251 pathways were enriched in the foregut compared to the midgut, 338 in the foregut compared to the hindgut, and 334 in the midgut compared to the hindgut. These pathways encompassed various biological processes, including 10 pathways related to the digestive system (e.g., Vitamin digestion and absorption, Bile secretion, Mineral absorption, Protein digestion and absorption), 23 pathways related to the Endocrine system (e.g., Renin-angiotensin system, PPAR signaling pathway), 22 pathways related to the Immune system (e.g., Hematopoietic cell lineage, Antigen processing and presentation, Th1 and Th2 cell Differentiation), 14 pathways related to Lipid metabolism (e.g., Fatty acid elongation, Arachidonic acid metabolism, Steroid hormone biosynthesis), seven pathways related to Transport and catabolism (e.g., Lysosome, Peroxisome, Endocytosis), and 267 pathways related to various other biological systems (Figure 5).

### 3.4. Expression of Representative DEGs Related to Nutrient Digestion and Absorption

**Figure 6:**
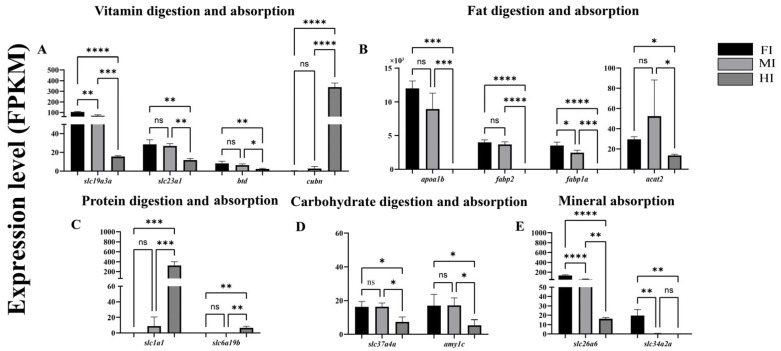
Differentially expressed genes (DEGs) associated with the digestion and absorption of sugars, proteins, fats, and inorganic salts in different intestinal segments of *S. dumerili* are presented (log_2_ (fold change, FC) > 1 and false discovery rate, FDR ≤ 0.05). The data are derived from fragments per kilobase of transcript per million mapped reads (FPKM) values obtained through RNA sequencing (RNA-—seq) analysis. (**A**): Genes associated with vitamin digestion and absorption; (**B**): Genes associated with fat digestion and absorption; (**C**): Genes associated with protein digestion and absorption; (**D**): Genes associated with carbohydrate digestion and absorption; (**E**): Genes associated with mineral absorption. *, *p* ≤ 0.05; **, *p* ≤ 0.01; ***, *p* ≤ 0.001,****, *p* ≤ 0.0001; ns, *p* ≥ 0.05.

Transcriptome data revealed distinct expression profiles of 14 representative DEGs involved in nutrient absorption and digestion (Figure 6). Genes related to vitamin digestion and absorption (*slc19a3a*, *slc23a1*, *btd*) were significantly upregulated in the foregut and midgut compared to the hindgut, except for *cubn*, which showed the highest expression in the hindgut (Figure 6A). Similarly, genes associated with lipid digestion and absorption (*apoa1b*, *fabp1a*, *fabp2*, *acat2*) exhibited higher expression in the foregut and midgut than in the hindgut (Figure 6B). Conversely, genes linked to protein digestion and absorption (*slc1a1*, *slc6a19b*) were significantly upregulated in the hindgut (Figure 6C). Genes involved in carbohydrate digestion and absorption (*slc37a4*, *amy1c*) showed higher expression in the foregut and midgut (Figure 6D), while those related to mineral digestion and absorption (*slc26a6*, *slc34a2*) were predominantly expressed in the foregut (Figure 6E).

### 3.5. Analysis of Microbiota Diversity in Different Gut Segments

MiSeq sequencing generated 157,646 optimized sequences, with 49,331, 52,064, and 56,251 effective reads in the foregut, midgut, and hindgut, respectively. Cluster analysis identified 782, 393, and 391 operational taxonomic units (OTUs) in the foregut, midgut, and hindgut, respectively. Among these, 196 OTUs were common to all three segments, while 471, 115, and 116 OTUs were exclusive to the foregut, midgut, and hindgut, respectively. Alpha diversity analysis results for different intestinal segments of *S. dumerili* are summarized in Table 2. While no significant difference was observed in Shannon’s index across the segments (*p ≥* 0.05), Chao’s index and ACE index were notably higher in the foregut compared to the midgut and hindgut (*p ≤* 0.05). The species richness of the microbiota in the foregut was significantly greater than in the midgut and hindgut, while the species evenness was lower in the foregut. Simpson’s index and Pielou’s evenness index indicated that the microbiota homogeneity was lowest in the foregut, with similar levels in the midgut and hindgut. The PD-whole tree index further highlighted that gut microbiota diversity was highest in the foregut and lowest in the hindgut.

### 3.6. Structural Composition of the Gut Microbiota

**Figure 7:**
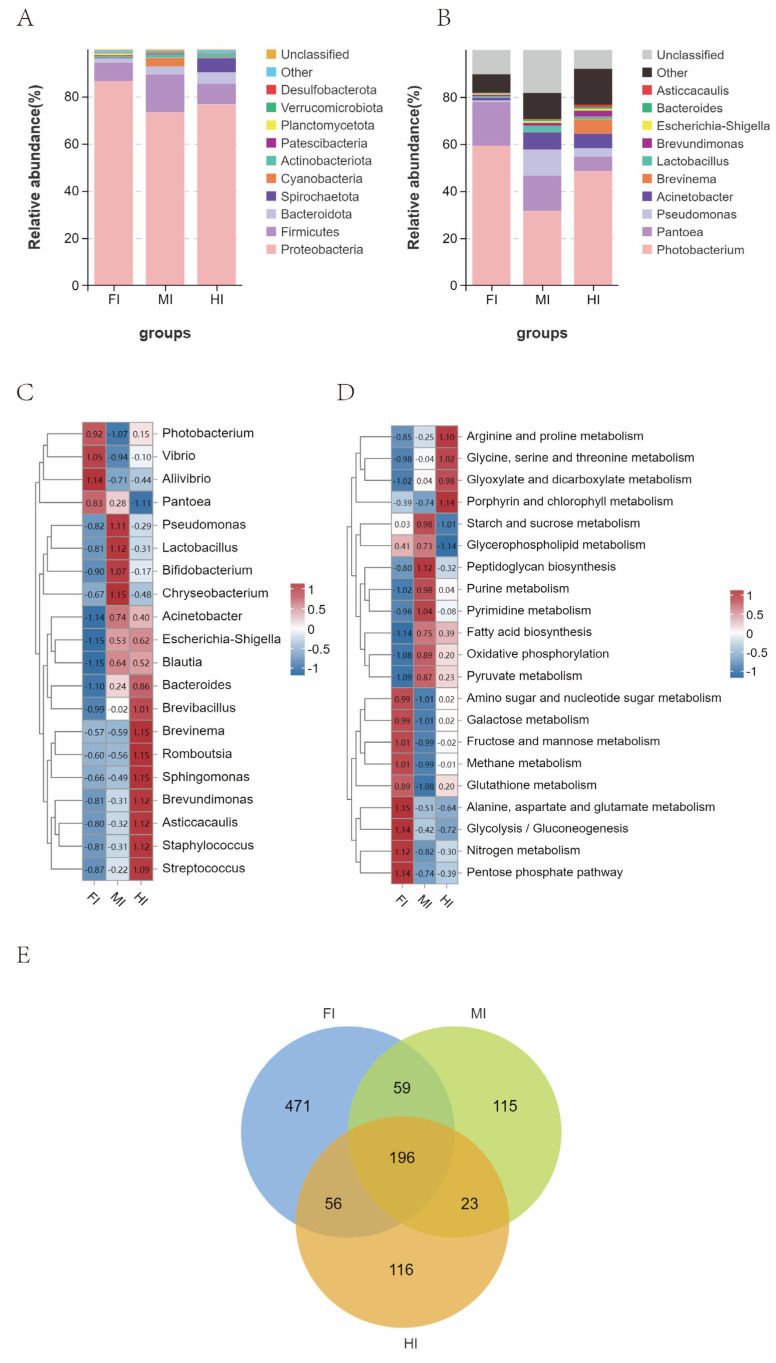
Changes in the relative abundance of gut microbes in different gut segments of *S. dumerili*. (**A**): Stacked species distribution at the phylum level; (**B**): Stacked species distribution at the genus level; (**C**): Heat map displaying species abundance at the genus level; (**D**): Heat map of Tax4Fun KEGG function prediction; (**E**): Venn diagram illustrating gut microbial classification across the different gut segments of *S. dumerili*.

The predominant bacterial phyla in *S. dumerili* included *Proteobacteria*, *Firmicutes*, *Bacteroidota*, *Spirochaetota*, and *Cyanobacteria*, with varying relative abundances across segments (Figure 7A). For instance, *Cyanobacteria* were more abundant in the midgut, while *Spirochaetes* were enriched in the hindgut. The *Firmicutes*/*Bacteroidota* (F/B) ratio decreased progressively from the foregut (4.398) to the hindgut (1.877).

At the genus level, Photobacterium dominated all segments, with secondary genera including *Pantoea* and *Acinetobacter* in the foregut, *Pantoea* and *Pseudomonas* in the midgut, and *Acinetobacter* and *Pantoea* in the hindgut (Figure 7B). Significant differences in bacterial genera abundance were observed across segments, with *Photobacterium*, *Vibrio*, *Pantoea* and *Alivibrio* enriched in the foregut, *Pseudomonas*, *Chryseobacterium*, *Lactobacillus* and *Bifidobacterium* in the midgut, and *Brevinema*, *Romboutsia*, *Brevundimonas* and *Sphingomonas* in the hindgut (Figure 7C).

KEGG functional analysis identified 266 significantly altered pathways (Table S3). Notably, pathways related to alanine, aspartate and glutamate metabolism, fructose and mannose metabolism, Glycolysis/Gluconeogenesis, Pentose phosphate pathway, and Nitrogen metabolism pathways were upregulated in the foregut. In the midgut, Starch and sucrose metabolism, Peptidoglycan biosynthesis, Purine metabolism, and Pyrimidine metabolism pathways were significantly upregulated. Finally, in the hindgut, pathways such as Arginine and proline metabolism, Glycine, serine and threonine metabolism, Glyoxylate and dicarboxylate metabolism, and Porphyrin were notably upregulated (Figure 7D).

### 3.7. Validation of DEGs with qRT-PCR

**Figure 8:**
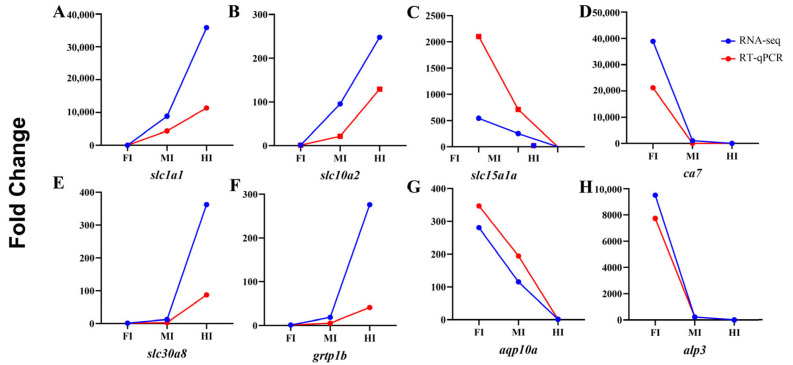
Validation of the expression of four upregulated and four downregulated genes using qRT-PCR.

To validate the RNA-seq results, four upregulated (*slc1a1*, *slc10a2*, *grtp1b*, *slc30a8*) and four downregulated (*ca7*, *slc15a1a*, *aqp10a*, *alp3*) genes were randomly selected for qRT-PCR analysis. The expression levels of these genes were consistent with the RNA-seq data, confirming the reliability of the transcriptome results (Figure 8).

## 4. Discussion

### 4.1. Structural and Functional Zonation of Intestinal Segments

Although intestinal folding is a common feature among many species, *Seriola dumerili* exhibits a distinct folding organization that aligns with its carnivorous feeding strategy [44,45]. Recent studies have highlighted the adaptive significance of intestinal morphology in teleosts, particularly in species with specialized diets. For instance, carnivorous fish species like yellowtail (*Seriola quinqueradiata*) [46] develop highly convoluted intestinal folds to enhance nutrient absorption efficiency for protein-rich diets. Similarly, the intestinal folding pattern in Atlantic cod (*Gadus morhua*) [47] correlates with its prey composition, showing increased surface area in regions specialized for lipid digestion. In *Seriola dumerili*, the pronounced folding height in the foregut and hindgut (Table 1) likely reflects its adaptation to digesting high-lipid prey, consistent with the functional specialization observed in other carnivorous teleosts. These structural features are indispensable for the digestive and absorptive functions of the intestine [48]. The foregut and hindgut exhibit significantly longer villi than the midgut, a morphological feature that likely enhances the intestinal surface area for efficient nutrient absorption. While peristaltic movements are primarily regulated by the thickness of the muscularis propria (with the foregut showing the thickest muscle layer), the extended villi in these segments create a specialized microenvironment to maximize contact between chyme and epithelial cells, facilitating lipid and protein absorption consistent with their carnivorous diet [2,48]. This morphological distinction enables stronger peristaltic forces in the foregut, ensuring optimal contact between food and epithelial cells, while the increased surface area in the hindgut enhances interactions with chyme and food residues, promoting efficient nutrient absorption and fecal consolidation. Such segmental specialization aligns with adaptive strategies in carnivorous teleosts, such as lipid-dominant foregut digestion in European seabass [8] and hindgut protein absorption in tambaqui [9], highlighting conserved functional zonation across species. The midgut, with its intermediate brush border microvillus [44] length, likely serves a dual role in enzymatic hydrolysis and immune surveillance, a functional balance also noted in European seabass (*Dicentrarchus labrax*) under dietary modulation [8].

Mucous cells [49] and goblet cells [50,51] play a pivotal role in secreting essential components of intestinal mucus, including mucopolysaccharides, mucins, and glycoproteins [13,52]. These secretory cells are vital for intestinal lubrication, facilitating food passage, maintaining intestinal homeostasis, and supporting mucosal immune function, thereby acting as the primary defense barrier of the intestinal immune system [53]. The distribution of goblet cells in *Seriola dumerili* exhibits segment-specific patterns that align with functional specialization (Table 1). The midgut, with the highest density of goblet cells, predominantly contains Type III mucous cells (neutral mucopolysaccharides) and Type IV cells (acidic mucopolysaccharides), which secrete mucus rich in glycoproteins and mucins. This robust secretion likely supports the midgut’s role as the primary site for carbohydrate digestion [5] (highest α-amylase activity, Figure 3B) and immune defense (elevated SOD, GSH-Px, and T-AOC, Figure 3G–I), as mucus provides a physical barrier against pathogens while facilitating enzyme-macromolecule interactions [49]. In the foregut, fewer goblet cells but diverse types (I–IV) may balance mechanical protection during food transit with lipid digestion (highest lipase activity, Figure 3A). The hindgut, with the lowest goblet cell density and limited types (II–III), suggests a reduced need for mucus-mediated immune surveillance, consistent with its role in protein digestion and fecal consolidation. Higher mucus secretion in the foregut and midgut is likely associated with mucosal protection against mechanical stress and pathogen invasion, whereas mucus production in the hindgut may play a more significant role in modulating microbial communities [54]. This spatial variation in mucus cell density underscores the functional compartmentalization of the intestine, reflecting evolutionary adaptations to dietary and environmental demands. While this study identifies four mucous cell types via AB-PAS staining, quantitative analysis of individual types was limited by our team’s preliminary experience with histochemical techniques. Future work will employ higher-resolution microscopy and standardized staining protocols to resolve subtype-specific distributions.

### 4.2. Digestive Enzyme Activities in Different Intestinal Segments

In fish, the intestine is the primary site for nutrient digestion and absorption, where digestive enzymes break down macromolecular nutrients such as proteins, lipids, and carbohydrates into smaller, more absorbable molecules like amino acids, fatty acids, and monosaccharides [13,52]. The three key enzymes involved in this process are α-amylase, lipase, and protease, which catalyze the hydrolysis of carbohydrates into monosaccharides, fats into fatty acids, and proteins into amino acids, respectively [13].

In this study, the low α-amylase activity in *S. dumerili* aligns with its carnivorous diet, which is naturally low in starch—a trait consistent with other obligate carnivorous teleosts like yellowtail (*Seriola quinqueradiata*) [46] and Atlantic cod (*Gadus morhua*) [47], where α-amylase activity is also restricted to the midgut and correlated with minimal carbohydrate intake. By contrast, omnivorous species such as common carp (*Cyprinus carpio*) [13] and Southern catfish (*Silurus meridionalis*) [55] exhibit two- to threefold higher midgut α-amylase activity, reflecting their reliance on plant-derived carbohydrates. Notably, while *S. dumerili* shows limited carbohydrate digestive capacity, its foregut lipase activity surpasses that of both midgut and hindgut by 1.5 to two times (Figure 3A), a feature shared with other lipid-rich diet specialists like European seabass (*Dicentrarchus labrax*) [8], where foregut hypertrophy and elevated lipase expression facilitate efficient lipid hydrolysis. This functional zoning—with the foregut prioritizing lipid digestion and the midgut retaining modest carbohydrate processing—represents an adaptive strategy to maximize nutrient extraction from protein- and lipid-dense prey, distinct from the more generalized digestive profiles of omnivorous species. Conversely, protease activity peaked in the midgut, highlighting its importance in protein hydrolysis. These findings suggest a clear functional zoning along the intestinal tract, with the foregut primarily responsible for lipid digestion and the midgut for protein and carbohydrate processing. Additionally, AKP and ACP, enzymes involved in the metabolism of glucose, calcium, and inorganic phosphorus, exhibited higher activity in the foregut and midgut compared to the hindgut. This spatial distribution implies that the foregut and midgut are the main sites for the digestion and absorption of inorganic salts. The hindgut, with its lower enzymatic activity, appears to play a more limited role in nutrient processing, consistent with its primary function in fecal consolidation and water reabsorption.

These results are supported by previous studies [56] and reinforce the concept of functional compartmentalization in the fish intestine. The absence of retrograde enzyme movement during chyme passage further underscores the spatial segregation of digestive functions along the intestinal tract [57]. Such specialization likely reflects evolutionary adaptations to optimize nutrient extraction and energy efficiency in *S. dumerili*, a carnivorous species with distinct dietary requirements.

### 4.3. Immune-Related Indicators in Different Intestinal Segments

SOD, GSH-PX, T-AOC, MDA, ACP, and AKP are key biomarkers for assessing antioxidant and immune responses in fish. SOD is an enzyme that catalyzes the dismutation of superoxide anion free radicals (O_2−_) to H_2_O_2_ and O_2_ [58], playing a critical role in neutralizing reactive oxygen species (ROS) and maintaining intracellular redox balance [49]. GSH-Px metabolizes dismutated O_2_ and H_2_O_2_ products by oxidizing reduced glutathione (GSH) to its oxidized form. A decrease in GSH-Px enzyme activity indicates a decline in the body’s ROS scavenging ability and antioxidant capacity [59,60]. T-AOC represents the total antioxidant level, comprising various antioxidants and enzymes like vitamin C, vitamin E, and carotenoids. ACP and AKP are important non-specific immune enzymes in the body. ACP and AKP, while primarily involved in phosphate metabolism, also contribute to non-specific immune responses by hydrolyzing foreign phosphate esters and modulating calcium-phosphorus homeostasis [61]. MDA, a byproduct of lipid peroxidation, serves as a marker of oxidative damage, inversely correlating with overall antioxidant capacity [62].

In this study, the midgut emerged as the primary site for antioxidant and immune activity, exhibiting significantly higher SOD, GSH-Px, and T-AOC levels compared to the foregut and hindgut. This elevated antioxidant capacity likely counterbalances ROS generated during nutrient processing and microbial interactions, which are particularly intense in the midgut due to its role in enzymatic hydrolysis and nutrient absorption. The midgut’s structural complexity—characterized by dense mucosal folds and abundant mucus secretion—creates an antigen-trapping microenvironment that facilitates localized immune responses through gut-associated lymphoid tissue (GALT). These findings align with the functional specialization of carnivorous teleosts, yet contrast with species like the Amur catfish (*Silurus asotus*), where the hindgut serves as the primary immune site, supported by thickened muscular layers and elevated goblet cell density [14]. In *S. dumerili*, the midgut’s role as an immune hub—marked by high SOD (higher than foregut, Figure 3G) and GSH-Px activity (higher than hindgut, Figure 3I)—likely stems from its structural complexity, including dense mucosal folds and the highest mucus cell density (Table 1), which create an ideal microenvironment for immune surveillance. Comparative studies in omnivorous species, such as common carp (*Cyprinus carpio*) [13], reveal a more dispersed immune response, with SOD and GSH-Px activities distributed across all intestinal segments. Even within carnivores, differences exist: Atlantic salmon (*Salmo salar*) [63,64] exhibits elevated immune enzyme activity in the hindgut, possibly linked to its pelagic feeding habits, whereas *S. dumerili*’s midgut dominance reflects adaptation to benthic prey rich in potential pathogens. The midgut’s low MDA levels (lower than hindgut, Figure 3F) further indicate reduced oxidative stress, a trait also reported in the midgut of lipid-rich diet specialists like European seabass (*Dicentrarchus labrax*) [8], where mucosal integrity is critical for sustained nutrient absorption.

### 4.4. Differential Expression of Digestion and Absorption Related Genes in Different Intestinal Segments of S. dumerili

The digestion and absorption of nutrients, including proteins, lipids, carbohydrates, minerals, and vitamins, are regulated by a complex interplay of genes and enzymes within the intestine. One key gene involved in amino acid absorption is *slc6a19b*, which encodes a transporter critical for the uptake of small molecule amino acids [56]. In *Ctenopharyngodon Idella*, reduced expression of *slc6a19b* has been shown to impair amino acid absorption capacity [65]. This finding aligns with carnivorous teleosts like yellowtail (*Seriola quinqueradiata*) [46,66] and Atlantic cod (*Gadus morhua*) [47,67,68], where hindgut-specific upregulation of amino acid transporters (*slc6a19*) correlates with efficient protein digestion from prey-rich diets. In contrast, omnivorous species such as common carp (*Cyprinus carpio*) [13,69] exhibit higher slc6a19 expression in the midgut, reflecting their more generalized digestive strategy with mixed protein-carbohydrate intake. The hindgut’s role in *S. dumerili* is further supported by its structural adaptations—thicker muscular layers (Table 1) and specialized villi—that enhance mechanical digestion and nutrient uptake, a trait also reported in the hindgut of other lipid-protein specialists like yellow catfish (*Pelteobagrus fulvidraco*) [70,71].

Lipid digestion and absorption are mediated by genes such as *apoa1b*, *acat2*, *fabp1a*, and *fabp2*. Apolipoprotein A4 (APOA4), encoded by the *apoa1b* gene, is a major apolipoprotein involved in lipid transport and metabolism [59]. The enzyme product of the *acat2* gene participates in the absorption and metabolism of lipids, including cholesterol, in the intestine [60]. Members of the fatty acid binding protein family, Fabp1a and Fabp2, bind to long-chain fatty acids and bile acids in food, enhancing the uptake, metabolism, and transport of fatty acids [72]. In this study, *apoa1b*, *fabp1a*, and *fabp2* exhibited the highest expression levels in the foregut, whereas *acat2* was significantly upregulated in both the foregut and midgut compared to the hindgut. This specialization is conserved in obligate carnivores like yellowtail (*Seriola quinqueradiata*) [46,66,73] and Atlantic cod (*Gadus morhua*) [8], where foregut-specific upregulation of *apoa1b* and *fabp1a* correlates with efficient lipid emulsification and uptake from protein-rich diets. In contrast, omnivorous species such as common carp (*Cyprinus carpio*) [13] exhibit reduced foregut lipid transporter expression, with *fabp2* preferentially expressed in the midgut to process plant-derived lipids. The foregut’s dominant role in *S. dumerili* is further supported by its structural adaptations—thicker muscular layers and elongated villi (Table 1)—which enhance contact between dietary lipids and lipase-secreting epithelial cells, a trait also reported in the foregut of Atlantic cod (*Gadus morhua*) [47,67,68], where high *acat2* activity facilitates cholesterol absorption [4].

Carbohydrate metabolism is regulated by genes such as *slc37a4*, which facilitates glucose-6-phosphate transport from the cytoplasm to the endoplasmic reticulum, maintaining glucose homeostasis [70]. The highest expression of *slc37a4* in the midgut of *S. dumerili* aligns with carnivorous teleosts like pikeperch (*Sander lucioperca*) [74,75], where midgut-specific carbohydrate transporter genes are upregulated to process limited dietary carbohydrates from prey. In contrast, omnivorous species such as common carp (*Cyprinus carpio*) [13] exhibit broader midgut expression of *slc37a4* and *amy1c*, reflecting their higher reliance on plant-derived carbohydrates. This midgut dominance in *S. dumerili* is further supported by its structural features, including the highest mucus cell density (Table 1), which likely creates an optimal microenvironment for carbohydrate enzyme activity and nutrient absorption, distinct from the more generalized digestive profiles of non-carnivorous species.

Mineral absorption, particularly phosphorus, is regulated by *slc34a2a*, a member of the solute carrier family [76]. Studies in carnivorous yellow catfish (*Pelteobagrus fulvidraco*) [70] and grass carp (*Ctenopharyngodon idella*) [71] have shown that *slc34a2* expression peaks in the foregut, aligning with efficient phosphorus uptake from animal-derived diets. This pattern is conserved in *S. dumerili*, where foregut-specific *slc34a2* expression suggests a specialized role in mineral absorption, similar to other carnivorous teleosts like Atlantic cod (*Gadus morhua*) [67,68,77]. In contrast, omnivorous species such as southern catfish [55] exhibit higher *slc34a2* expression in the midgut, reflecting their adaptation to plant-based phosphorus sources that require extended digestive processing. The foregut’s dominance in *S. dumerili* is further supported by its structural adaptations—thicker muscular layers and elongated villi (Table 1)—which enhance contact with mineral-rich chyme, a trait shared by phosphorus-efficient carnivores.

Vitamin absorption is mediated by genes such as *btd* [78], *cubn* [79], *slc19a3* [80], and *slc23a1* [81]. Dysregulation of these genes can lead to impaired absorption and transport of specific vitamins. In this study, *btd* (linked to biotin absorption) and *slc19a3* (associated with vitamin B7 absorption) exhibited higher expression in the foregut and midgut, while *slc23a1* (involved in vitamin C absorption) was predominantly expressed in the foregut. In contrast, *cubn* (related to vitamin B12 absorption) showed the highest expression in the hindgut. These results indicate that while the foregut is the primary site for most vitamin absorption, certain vitamins, such as vitamin B12, may be predominantly absorbed in the hindgut of *S. dumerili*.

### 4.5. Gut Microbiota and Functional Specialization Across Intestinal Segments

The gut microbiota plays a pivotal role in maintaining intestinal health and facilitating nutrient metabolism in fish. The gut microbiota of *S. dumerili* exhibits striking segmental specialization, with functional profiles that align closely with host digestive and immune needs. In the foregut, the dominance of *Ruminococcus*—a genus associated with lipid β-oxidation and fatty acid metabolism—mirrors findings in other carnivorous teleosts like pikeperch (*Sander lucioperca*) [74], where similar microbial taxa enhance energy extraction from lipid-rich prey [82,83]. This microbial-enzymatic synergy likely optimizes lipid digestion, compensating for the host’s reliance on Carnivorous diets.

In contrast, the midgut was dominated by *Prevotella*, a genus within the Bacteroidetes phylum renowned for its ability to degrade complex polysaccharides and release energy from dietary fiber and starch [83]. The high prevalence of *Prevotella* in the midgut underscores its role as the primary site for carbohydrate metabolism, complementing the host’s limited endogenous capacity for starch digestion. This microbial niche specialization mirrors findings in other carnivorous fish, where midgut microbiota compensate for host enzymatic deficiencies [84]. The midgut also displayed the highest *Firmicutes*/*Bacteroidetes* (F/B) ratio, a metric closely associated with intestinal inflammation and immune regulation. A higher F/B ratio correlates with increased butyrate production, a short-chain fatty acid (SCFA) known to enhance anti-inflammatory responses and strengthen epithelial barrier integrity [83,85]. The midgut’s robust anti-inflammatory environment is further supported by the abundance of *Bifidobacterium*, *Lactobacillus*, and *Carnobacterium*, genera that produce organic acids, bacteriocins, and extracellular glycosidases to inhibit pathogenic colonization and maintain microbial equilibrium [86]. For instance, *Lactobacillus plantarum* has been shown to enhance disease resistance in *Epinephelus coioides* by modulating immune responses [87], while *Carnobacterium* spp. inhibit opportunistic pathogens like *Aeromonas salmonicida* and *Aeromonas hydrophila* in salmonids [88]. Additionally, *Phascolarcto bacterium*, a butyrate-producing genus, was enriched in the midgut, further contributing to its anti-inflammatory milieu [89]. In the midgut, the enrichment of *Prevotella* (polysaccharide degradation) and probiotic genera (*Bifidobacterium*, *Lactobacillus*) aligns with its dual role in carbohydrate metabolism and immune defense. Comparative studies in European seabass (*Dicentrarchus labrax*) [87] reveal that *Prevotella*-dominated midgut microbiota compensate for limited host α-amylase activity, a strategy conserved in carnivores with low dietary starch intake. Concurrently, the high *Firmicutes*/*Bacteroidetes* ratio in the midgut, linked to butyrate production by *Phascolarctobacterium*, mirrors anti-inflammatory mechanisms reported in Atlantic salmon (*Salmo salar L*.) [88], where short-chain fatty acids reinforce epithelial barrier integrity. The hindgut’s depauperate microbiota, dominated by *Brevinema* and *Sphingomonas*, reflects its minimal digestive role–a trait shared by carnivorous species like Atlantic cod (*Gadus morhua*) [90], where hindgut microbes primarily facilitate water reabsorption and fecal formation. This microbial zonation, from lipid-processing foregut to immune-competent midgut and absorptive hindgut, represents an evolutionary adaptation to maximize nutrient utilization while minimizing energy expenditure on non-essential functions.

These findings underscore the intricate interplay between microbial ecology and host physiology in *S. dumerili*. The spatial variation in microbiota composition reflects functional compartmentalization, with the foregut specializing in lipid digestion, the midgut in carbohydrate metabolism and immunity, and the hindgut in waste management. This microbial zonation not only optimizes nutrient utilization but also enhances disease resistance, providing valuable insights for improving aquaculture practices and feed formulations.

## 5. Conclusions

The intestine of *Seriola dumerili* exhibits a remarkable functional zonation despite its morphologically conserved structure, as demonstrated by integrated histological, enzymatic, transcriptomic, and microbiome analyses. Histological observations revealed that the foregut and hindgut possess thicker muscular layers and longer villi, adapting to mechanical digestion and nutrient absorption, while the midgut harbors the highest density of goblet cells and immune enzyme activities, positioning it as the core site for mucosal immunity and carbohydrate metabolism. Transcriptome sequencing and microbiota profiling further validated this specialization: the foregut prioritizes lipid digestion via *apoa1b* and *Ruminococcus*-dominated microbes, the midgut integrates carbohydrate metabolism (*slc37a4*) with immune surveillance (*Bifidobacterium* enrichment). and the hindgut specializes in protein absorption (*slc6a19b*) with simplified mucus secretion. These findings establish a clear framework for segment-specific functions, bridging structural adaptations with molecular and microbial mechanisms.

While this study provides a foundational understanding of intestinal specialization in carnivorous fish, future research should address three key frontiers: (1) Functional validation through multi-omics integration, such as combining transcriptomics with metabolomics to trace nutrient flux across segments or using gnotobiotic models to dissect host-microbe interactions identified here (e.g., midgut *Lactobacillus*–mucus cell crosstalk); (2) Environmental and dietary modulation, exploring how factors like water temperature, feed formulation (e.g., plant protein inclusion), or probiotic supplementation (e.g., *Bifidobacterium* strains) influence intestinal zonation, as hinted by the midgut’s sensitivity to microbial changes; (3) Cell-type specificity, leveraging single-cell RNA sequencing to resolve rare cell populations (e.g., enteroendocrine cells in the hindgut) and their roles in nutrient sensing and immune signaling, which were under resolved by bulk transcriptomics (n = 3 per segment). Such advancements will not only deepen our mechanistic understanding of fish intestinal physiology but also inform precision aquaculture strategies, from designing segment-targeted diets to developing probiotics that enhance mucosal health in *S. dumerili* and related carnivorous species. By linking basic biology to applied nutrition, this research paves the way for sustainable improvements in fish health, growth, and resource efficiency in modern aquaculture systems.

## Figures and Tables

**Table 1:** Intestine of different intestinal segments in *S. dumerili*.

Indices	Foregut	Midgut	Hindgut
Brush border microvillus length (µm)	27.89 ± 4.60 ^a^	22.01 ± 4.53 ^b^	28.23 ± 5.36 ^a^
Muscular thickness (µm)	289.49 ± 30.40 ^a^	167.28 ± 14.41 ^c^	239.30 ± 15.24 ^b^
Mucosal folds height (µm)	984.51 ± 96.65 ^b^	865.85 ± 58.11 ^b^	1182.01 ± 111.44 ^a^
Total mucus cell	133.17 ± 10.07 ^b^	166.5 ± 9.97 ^a^	32.33 ± 3.14 ^c^

Note: Different letters (a, b, c) denote statistically significant differences between the segments (*p* < 0.05). Total mucous cell counts include all four types (I–IV) identified in Figure 2.

**Table 2:** Diversity index of microbiota in different intestinal segments of *S. dumerilis*.

Group	Shannon	Simpson	Chao	ACE	Pielou	Pd
FI	2.47	0.52	760.63	766.36	0.27	168.49
HI	3.25	0.64	553.44	599.86	0.38	48.52
MI	3.10	0.69	567.81	605.70	0.36	90.37

## Data Availability

The raw RNA sequencing data has been submitted to the Sequence Read Archive (SRA) database (http://www.ncbi.nlm.nih.gov/sra/) (accessed on 14 June 2023) under Bioproject number PRJNA1069331.

## Supplementary Materials

The following supporting information can be downloaded at: https://www.mdpi.com/article/10.3390/ani15111672/s1, Table S1: Primer sequences used in this study. Table S2. Statistics of genes identified in the different libraries. Table S3. Results of species distribution stacking at the Genus level in this study (Retain species with >0.1% abundance in at least 1 sample).
